# Efficacy and Mechanism of Acupoint Catgut Embedding in the Treatment of Chronic Spontaneous Urticaria: Protocol for a Randomized Double-Blind Placebo-Controlled Trial

**DOI:** 10.2196/54376

**Published:** 2024-07-31

**Authors:** Jianing Bi, Li Liu, Zhu Fan, Shengyuan Qu, Jiao Yang, Chenchen Xu, Bingnan Cui

**Affiliations:** 1 Beijing University of Chinese Medicine Beijing China; 2 Guang'anmen Hospital China Academy of Chinese Medical Sciences Beijing China

**Keywords:** chronic spontaneous urticaria, acupoint catgut embedding, functional magnetic resonance imaging, randomized controlled trial, mechanism of action

## Abstract

**Background:**

Chronic spontaneous urticaria (CSU) is a common chronic inflammatory skin disease that manifests as itching and wheals, seriously affecting quality of life. Clinical observations and previous research trials have shown that acupuncture is safe and effective for the treatment of CSU. However, there are problems, such as a short duration of action and frequent treatment. Compared with traditional acupuncture, acupoint catgut embedding (ACE) has the advantages of a longer effect and higher compliance. Clinical trials are needed to prove its efficacy and mechanism of action.

**Objective:**

This trial aims to provide definitive evidence for the treatment of CSU with ACE and explore the mechanism of ACE.

**Methods:**

This is a randomized, double-blind, placebo-controlled trial. In this trial, 108 participants aged 18-60 years with a diagnosis of CSU and no history of ACE will be randomly assigned to 2 groups (1:1 ratio) using the Statistical Analysis System: treatment (ACE) and control (sham ACE). The participants and efficacy evaluators will be blinded to the grouping. Both the ACE and sham ACE groups will undergo acupuncture, but the sham ACE group will not receive catgut sutures. Treatment will be performed twice weekly for 8 weeks, with a 1-week run-in period and a 16-week follow-up period. Twenty patients will be randomly selected to undergo functional magnetic resonance imaging before and after the treatment period. The primary outcome will be the urticaria activity score over 7 days (UAS7). We will use R (version 4.0.1; R Project for Statistical Computing) to perform ANOVA and independent samples *t* tests to compare the differences within and between groups before and after treatment by judging the rejection range based on a significance level of .05.

**Results:**

The study protocol has been approved by the Ethics Committee of Guang’anmen Hospital on September 7, 2022, and has been registered on November 30, 2022. Recruitment began on March 1, 2023. A total of 4-6 participants are expected to be recruited each month. The recruitment is planned to be completed on March 1, 2025, and we expect to publish our results by the winter of 2025. As of November 1, 2023, we have enrolled 25 participants with CSU.

**Conclusions:**

This randomized, double-blind, placebo-controlled trial aims to provide definitive evidence for the treatment of CSU with ACE and explore the mechanism of ACE. We hypothesize that wheals and itching will show greater improvement in participants receiving active therapy than in those receiving sham treatment. The limitations of this study include its single-center trial design, small sample size, and short treatment duration, which may have certain impacts on the research results.

**Trial Registration:**

Chinese Clinical Trial Registry ChiCTR2200066274; https://www.chictr.org.cn/showprojEN.html?proj=179056

**International Registered Report Identifier (IRRID):**

DERR1-10.2196/54376

## Introduction

Chronic spontaneous urticaria (CSU) is a common chronic inflammatory skin disease that accounts for 90% of all chronic urticaria cases. It manifests as itching and wheals with continuous or intermittent attacks for more than 6 weeks. Angioedema accompanies 40% of cases of CSU, with 10% primarily presenting with angioedema [1,2]. CSU has no clear inducing factors, and a single episode usually disappears within 24 hours. The majority of individuals with this condition experience a disease duration of 2 to 5 years, often with self-limiting tendencies. However, in 20% of cases, the disease persists for more than 5 years, and 1.4% endure it throughout their life [3,4].

The pathogenesis of CSU is complex, and currently, it is mainly thought to be related to 2 types of autoimmune reactions: type I autoimmune reaction, which is caused by immunoglobulin E, and type IIb autoimmune reaction, which is caused by immunoglobulin G autoantibodies [5]. Recent studies have shown that factors, such as skin mast cell degranulation [6,7], basophil activation [8], and other serological factors, can also induce vasodilation, increase vascular permeability, stimulate sensory nerve endings, and lead to skin swelling, redness, and pruritus. However, the specific pathogenesis of CSU remains unclear. When considering patients who develop the disease without the influence of external triggers, it becomes evident that the primary factor at play is the disruption of internal regulatory mechanisms within the human body. Clinical observations have found that the psychological factors of patients, such as anxiety and depression, have an impact on the onset of urticaria [9]. CSU is characterized by repeated attacks that are prolonged and difficult to cure, which seriously affect the quality of life of patients [10], bring mental and psychological pressure to patients, and further aggravate the occurrence of CSU [11], forming a vicious circle.

Currently, there are no effective radical treatments for CSU. First-line treatment revolves around standard doses of second-generation H1 antihistamines. Second-line treatment involves combinations of medications, often in high doses. Third-line treatment primarily consists of cyclosporine and biologic agents [3], which have problems such as long drug cycles, easy recurrence of drug withdrawal, and obvious side effects. Traditional Chinese medicine (TCM) is widely used as a complementary and alternative therapy to treat CSU [12].

Research has confirmed that the skin is both an immediate stress perceiver and a target of stress responses [13]. The psychological stress resulting from depression, anxiety, and other illnesses can ultimately lead to the development of CSU by exerting its effects on the skin through intricate neural systems. These systems include the activation of neuropeptides and neurokinins, the mediation of inflammatory mediators and cells, and the regulation of hypothalamic-pituitary-adrenal (HPA) axis hormones [14]. Neural metabolites or neurotransmitters released by the brain cause changes in skin function through the HPA axis. Abnormalities in the HPA axis can stimulate mast cells, which are the main effector cells of CSU, to release histamine. In addition, brain-derived neural metabolites can mediate or enhance skin inflammation.

In the TCM theory, all painful and itching sores are ascribed to the heart, which stores the spirit. This is consistent with the modern medical theory of urticaria and its associated psychological factors. The TCM theory can be integrated with modern medical research. TCM frequently uses treatments like herbal medicine and acupuncture in the clinical management of CSU. These treatments aim to nourish the heart, soothe the spirit, purge heart fire, and alleviate feelings of anxiety and depression. However, some patients may experience adverse reactions to herbal medicine, and the bitter taste of the medicine may intensify these reactions. These reactions can lead to resistance and increased psychological stress, potentially exacerbating their condition. Consequently, it is recommended to explore alternative therapies, such as acupuncture, as soon as possible.

In 2018, we selected acupoints related to the heart channel and conducted a randomized, double-blind, placebo-controlled clinical observational study titled “Treatment of CSU by acupuncture from the heart.” We enrolled 60 subjects and divided them randomly into 2 groups (a “treatment group” that received acupuncture and a “control group” that received oral loratadine only) for a 6-week study of acupuncture treatment performed 3 times weekly. As the treatment duration progressed, both groups exhibited improvements, with a significant reduction in the urticaria activity score over 7 days (UAS7). In addition, we used functional magnetic resonance imaging (fMRI) to reveal the brain mechanisms of CSU, and acupuncture was found to alleviate itch through a cerebellar reward-somatosensory circuit for the first time [15,16]. However, traditional acupuncture therapy has a short action time, a long treatment cycle, and many operations, which means that patients require frequent medical attention. This reduces patient compliance. Compared with traditional acupuncture, acupoint catgut embedding (ACE) has the advantages of a longer effect and higher compliance; however, there is a lack of high-quality clinical studies.

The main purpose of this research is to verify the efficacy and safety of ACE in the treatment of CSU based on the theoretical principle that “all pain and itchy sores belong to the heart.” Additionally, fMRI is used to reveal the central mechanism of ACE during CSU treatment.

## Methods

### Study Design

**Figure 1:**
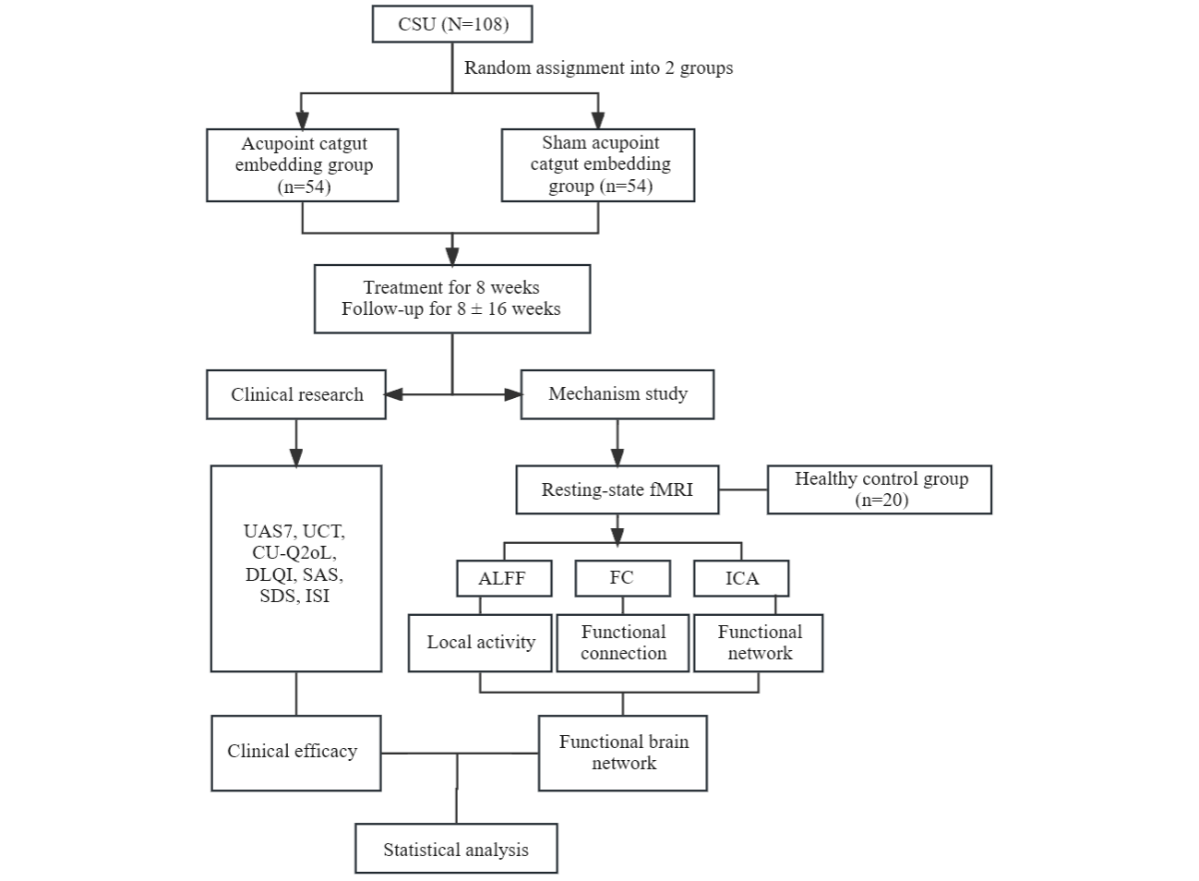
Flowchart illustrating the randomized, placebo-controlled, double-blinded trial of acupoint catgut embedding for CSU. ALFF: amplitude of low-frequency fluctuation; CSU: chronic spontaneous urticaria; CU-Q2oL: Chronic Urticaria Quality of Life Questionnaire; DLQI: Dermatology Life Quality Index; FC: functional connectivity; fMRI: functional magnetic resonance imaging; ICA: independent component analysis; ISI: Insomnia Severity Index; SAS: Self-Rating Anxiety Scale; SDS: Self-Rating Depression Scale; UAS7: urticaria activity score over 7 days; UCT: urticaria control test.

This study is designed as a 2-arm, randomized, placebo-controlled trial with both patients and evaluators blinded to group allocation. The study is divided into 3 phases: a 1-week run-in period, an 8-week intervention period, and a 16-week follow-up period. A total of 108 patients will be randomized into 2 groups (ACE and sham ACE groups), with 54 patients in each group. In each group, 20 patients will be selected to participate in fMRI before and after the treatment period. Additionally, we will recruit an age- and sex-matched control group of 20 healthy participants, who will undergo fMRI only before and after the treatment period. A flowchart of the trial is shown in Figure 1.

### Eligibility Criteria

#### Inclusion Criteria

The inclusion criteria are as follows: (1) age between 18 and 60 years, regardless of sex; (2) meeting the diagnostic criteria for CSU; (3) wheals and itching occurring continuously for at least 6 weeks before enrollment (despite using H1 antihistamines; occurring at least once daily) and wheals and itching being the main symptoms (without severe systemic symptoms); (4) a UAS7 of ≥16 and a weekly pruritus score of ≥8; (5) no history of ACE; and (6) signing of informed consent and volunteering to participate in the study.

#### Exclusion Criteria

The exclusion criteria are as follows: (1) clear identification of the cause of chronic urticaria (eg, physical urticaria); (2) use of glucocorticoids, hydroxychloroquine, methotrexate, cyclosporine, cyclophosphamide, or intravenous immunoglobulins for any indication (daily or more than 3 consecutive days) within the first 30 days of enrollment; (3) use of H2 antihistamines, leukotriene receptor antagonists, omalizumab, or other biological agents in the 7 days preceding screening or 14 days before randomization; (4) exceeding the recommended dosage of H1 antihistamines within 3 days before screening; (5) presence of serious cardiovascular and cerebrovascular diseases, diabetes, abnormal bone metabolism, liver and kidney diseases, hematopoietic system diseases, systemic lupus erythematosus, other serious autoimmune diseases, other diseases, and other serious primary diseases; (6) diseases other than chronic urticaria with symptoms of urticaria or angioedema, such as urticaria vasculitis, erythema multiform, cutaneous mastocytosis (pigmentary urticaria), and hereditary or acquired angioedema (eg, due to deficiency of C1 inhibitors); (7) skin diseases related to chronic pruritus (eg, atopic dermatitis, bullous pemphigus, herpetic dermatitis, and pruritus in old age), which may affect the evaluation and treatment in the study; (8) allergy to the buried wire material; (9) fertility needs, pregnancy, or lactation during the study period; (10) contraindicating magnetic resonance imaging (MRI) findings; (11) participation in other clinical trials within the last 3 months; and (12) use of psychotropic drugs within the previous month.

#### Inclusion Criteria for Healthy People

The inclusion criteria are as follows: (1) good health; (2) no serious physical illness and no family history of mental illness; (3) no history of alcohol or drug addiction; and (4) no contraindications for MRI scanning and no structural abnormalities in the brain.

#### Withdrawal Criteria and Management

Participants may be permitted or required to withdraw from the study for the following reasons: (1) experiencing serious adverse events or adverse events during the study period; (2) demonstrating poor compliance and showing a lack of cooperation with treatment and efficacy follow-up; (3) unauthorized use of nonclinical emergency drugs without information; (4) voluntarily requesting to withdraw from the trial; and (5) withdrawal from the trial before the end of the treatment course, loss to follow-up, or death for various reasons.

### Recruitment

The primary recruitment for this study will target outpatients from Guang’anmen Hospital in Beijing, China. In addition, recruitment efforts will include posters and online advertisements providing a concise overview of the trial along with the contact information of researchers. Before enrollment, all participants will receive a comprehensive briefing on the test procedures, study’s objectives, potential adverse events, and expected benefits. During the screening phase, each participant’s eligibility for inclusion will be assessed, and they will be explicitly informed of their right to withdraw from the trial at any point. Those who choose to participate in the trial will be required to sign 2 informed consent forms: one for their records and another to be retained by the researcher.

### Randomization, Concealment, and Blinding

Random numbers will be generated using the Statistical Analysis System PROC PLAN process statement to determine the number of seeds and the segment length. Treatment assignments corresponding to running numbers 001-108 will be listed. To ensure allocation concealment, the randomization process will be managed by an independent statistician who is not involved in the study. Eligible participants will be randomly assigned to the treatment or control group in a 1:1 ratio.

Random numbers will be sealed in an opaque envelope, which will be stored in the study center. To ensure the objectivity of the research, operators and efficacy evaluators will be strictly distinguished, and efficacy evaluators and statisticians will be blinded. Only operators will have access to random numbers before treatment, and they will not be involved in the evaluation process. The selected acupoints are mainly on the lower limbs and back. The control group will also undergo acupuncture, which will ensure patient blinding. Two-stage unblinding will occur only at the end of the case collection.

### Sample Size

Referring to relevant literature [17], a formula for the superiority test is used to calculate the sample size:



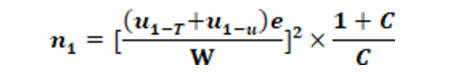





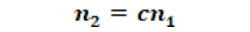



where *c*=1, *n*_2_=*n*_1_; if α=.05, *u*_1_-*T*=1.64 and *u*_1_-*u*=1.28.

According to previous research [15] based on the UAS7 as the main therapeutic evaluation index, *e* is set at 9.12, *W* is set at 6, and the data are substituted into the formula to calculate *n*_1_=*n*_2_≈40. Considering a 25% dropout rate, we establish a sample size of 54 cases in each group, with a total of 108 cases.

However, it is worth noting that when conducting MRI research on the human body, the calculation of sample size does not strictly adhere to the methods used in clinical trials. Currently, there are no clear guidelines for determining sample size in fMRI studies. After an extensive review of the literature spanning the past decade, we did not find a standardized sample size calculation method for experimental studies involving brain fMRI. The previous results of the research group showed that 20 cases in each group met the statistical and practical requirements [13,14]. In this study, 40 subjects are selected for fMRI examination based on the results of previous studies and the scale of the subjects.

### Interventions

#### ACE Approach

Both the ACE and sham ACE groups will undergo acupuncture at identical acupoints in the acupuncture treatment room at Guang’anmen Hospital. To investigate the potential for ACE to extend the therapeutic duration, the control group will not receive catgut sutures. The procedure involves the insertion of a 1.0-cm catgut suture into a hypodermic needle, which is then introduced into the acupoints through the skin [18]. The angle of the hypodermic needle will be selected according to the thickness of the muscle. The hypodermic needle will be withdrawn from the skin once the patient obtains qi.

**Figure 2:**
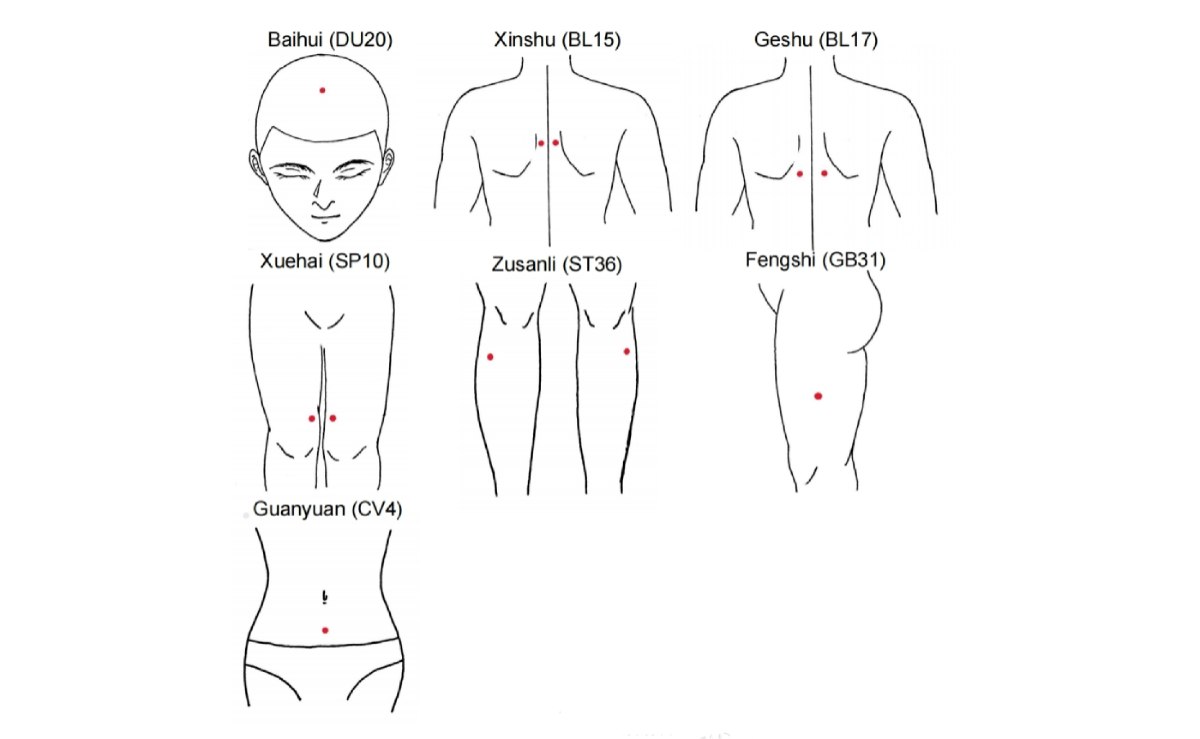
The locations of acupoints. DU: Du meridian; BL: bladder; SP: spleen; ST: stomach, GB: gall bladder; CV: conception vessel.

The treatment will be administered once every 2 weeks for a total of 8 weeks at the same 12 acupoints biweekly, including 4 acupoints on the dorsum, 6 acupoints on the lower limbs, 1 acupoint on the head, and 1 acupoint on the abdomen. The acupoints on the dorsum will be prone and others will be supine. These acupoints include Baihui (DU20), bilateral Xinshu (BL15), bilateral Geshu (BL17), Guanyuan (CV4), bilateral Xuehai (SP10), bilateral Zusanli (ST36), and bilateral Fengshi (GB31). The specific distribution of acupoints is provided in Table 1 and Figure 2. The precise locations of these acupoints and their Chinese pinyin names are according to acupuncture nomenclature established by the World Health Organization (WHO) [19]. The selection of these acupoints is based on a review of classical and modern literature, the neuroanatomical theory, and a consensus of acupuncture experts in Guang’anmen Hospital [20-22]. Twenty patients will be randomly selected from the ACE and sham ACE groups to undergo fMRI before and after the treatment period. For comparison, 20 healthy participants will be recruited to participate in fMRI before and after the treatment period without treatment.

The study will use sterile hypodermic needles produced by Jiangsu Zhenjiang Gaoguan Medical Equipment Co, Ltd, and 4-0’ catgut sutures produced by Jiangxi Longteng Biological Technology Co, Ltd.

**Table 1:** Locations and manipulation of acupuncture points.

Acupuncture point	Location	Suggested technique
Baihui (DU20)	On the head 5 cun (traditional Chinese unit of length) superior to the anterior hairline and on the anterior median line	Subcutaneous insertion (angle of 10°-15° and depth of 10-16 mm)
Xinshu (BL15)	In the lumbar region at the same level as the inferior border of the spinous process of the fifth thoracic vertebra (T5) and 1.5 cun lateral to the posterior median line	Perpendicular insertion (depth of 20-35 mm)
Geshu (BL17)	In the lumbar region at the same level as the inferior border of the spinous process of the seventh thoracic vertebra (T7) and 1.5 cun lateral to the posterior median line	Perpendicular insertion (depth of 20-35 mm)
Guanyuan (CV4)	On the lower abdomen 3 cun inferior to the center of the umbilicus and on the anterior median line	Perpendicular insertion (angle of 90° and depth of 20-35 mm)
Xuehai (SP10)	On the lower limbs 2 cun above the medial end of the patellar base	Perpendicular insertion (angle of 90° and depth of 20-35 mm)
Zusanli (ST36)	On the lower limbs 3 cun inferior to the outer knee and on the tibial edge	Perpendicular insertion (depth of 10-15 mm)
Fengshi (GB31)	On the lower limbs 7 cun superior to the popliteal transverse line	Perpendicular insertion (depth of 10-15 mm)

#### Concomitant Therapy

During the trial, only 1 H1 antihistamine can be taken. Other medicines or TCM with similar clinical efficacy, such as biological agents, H2 antihistamines, and hormones, must not be taken. If other therapies are combined, they will be recorded in the “Case Report Form (CRF).” If subjects require other treatments or concomitant care, they will need to contact the doctor in advance.

#### fMRI Examination

##### Pretest Preparation

The fMRI test will be conducted using a 3.0T Skyra (Siemens) MRI machine located at Guang’anmen Hospital, Chinese Academy of Medical Sciences. Following the completion of informed consent forms and subject checklists by the participants, the experimenter will explain the testing procedure and precautions. Prior to entering the scanning room, a thorough check will be conducted to ensure that there are no magnetic foreign objects on the participants’ bodies. Subsequently, the participants will be positioned in the supine posture on a scanning bed, and fMRI scans will be conducted under the conditions of quietude, closed eyes, and no specific cognitive activities. In the event of a rash attack, the scan will be postponed until the rash subsides for at least 12 hours.

##### Scanning Program and Parameters

A whole-brain transverse scan will be conducted in parallel with the anterior commissure-posterior commissure line and will include the cerebellum and brainstem. Resting-state functional magnetic resonance parameters will be as follows: time to repetition (TR), 2000 ms; time to echo (TE), 30 ms; flip angle (FA), 90°; layer thickness, 3.5 mm; layer spacing, 0.6 mm; 32 layers; field of view (FOV), 224×224 mm; interlayer resolution, 64×64; and scan time, 6 minutes and 46 seconds. A whole-brain 3D T1-weighted structural image will be captured through a 200-layer sagittal scan with the following parameters: layer thickness, 1.0 mm; no layer spacing; intralaminar resolution, 256×192; TR, 25300 ms; TE, 2.98 ms; FA, 7°; FOV, 256×256 mm; and scan time, 6 minutes and 3 seconds.

#### Adherence

To promote participant retention and ensure complete follow-up, the research team will initially emphasize the importance of subject compliance at the outset. The team will be in regular contact with the participants, checking on their progress once a week, providing reminders to remain engaged in treatment, and offering support throughout the treatment period. Following the completion of the full treatment regimen, participants will receive a subsidy as a token of appreciation. Participant adherence will be assessed by counting the number of treatments received, with the criterion of 80% or more of the prescribed treatments as the threshold for good adherence. The formula for calculating treatment adherence is as follows: treatment adherence = (number of treatments received by the subject / total number of treatments to be received by the subject) × 100.

### Outcomes

**Figure 3:**
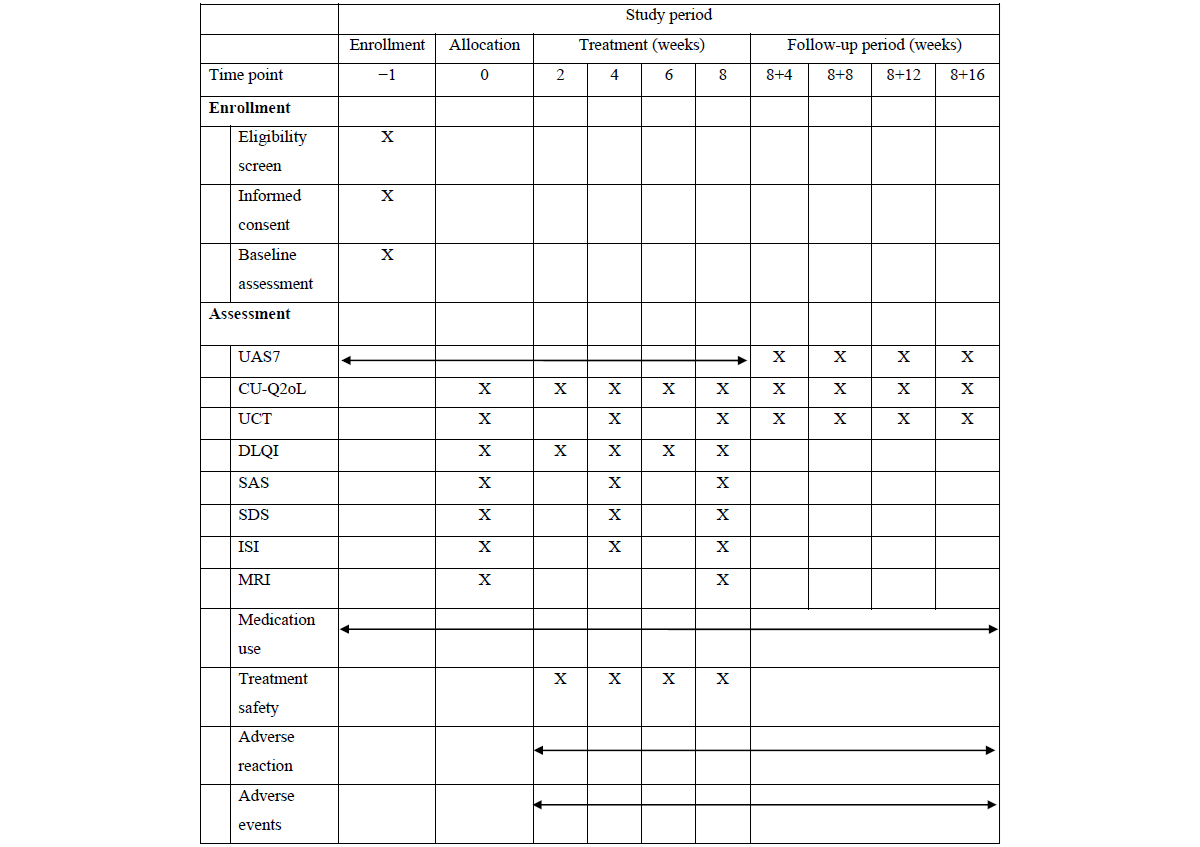
Schedule of trial enrollment, intervention, assessment, and follow-up. CU-Q2oL: Chronic Urticaria Quality of Life Questionnaire; DLQI: Dermatology Life Quality Index; ISI: Insomnia Severity Index; MRI: magnetic resonance imaging; SAS: Self-Rating Anxiety Scale; SDS: Self-Rating Depression Scale; UAS7: urticaria activity score over 7 days; UCT: urticaria control test.

The study schedule is presented in Figure 3. All outcome measures explained below will be recorded from baseline until the end of follow-up.

#### Primary Outcome

The primary outcome measure for this clinical trial will be the UAS7. The UAS7 is the gold standard for CSU, evaluating 2 skin symptoms: wheals and itching. Participants will self-assess and record the severity of their symptoms on a 4-point scale. For itchiness, the scoring will be as follows: 0, no symptoms; 1, mild symptoms (occasional itching, no scratching, and no effect on life and sleep); 2, moderate symptoms (occasional itching, scratching, and life and sleep slightly disturbed, but tolerable); 3, severe symptoms (frequent itching, scratching, life and sleep disturbed, and intolerable). For wheals, the scoring will be as follows: 0, no symptoms; 1, mild symptoms (<20 in 24 h); 2, moderate symptoms (21-50 in 24 h); 3, severe symptoms (>50 in 24 h or large confluent masses). The UAS7 ranges from 0 to 42, with lower scores indicating milder skin symptoms [23].

#### Secondary Outcomes

##### Quality of Life

The quality of life measures in this clinical trial will include the Chronic Urticaria Quality of Life Questionnaire (CU-Q2oL) [24] and the Dermatology Life Quality Index (DLQI) [25]. The CU-Q2oL comprises [25] questions that assess the physical, psychosocial, and everyday impact of CSU over the past 2 weeks. Meanwhile, the DLQI comprises 10 questions that evaluate how skin problems have affected the quality of life over the past week.

##### Urticaria Control Test

The urticaria control test (UCT) measures the level of disease control among patients with CSU over the past 4 weeks. It evaluates the level of distress caused by the physical symptoms of urticaria, the impact on QoL, the effectiveness of treatment in controlling urticaria symptoms, and the overall perceived level of urticaria control. Higher scores indicate better disease control.

##### Emotion

The emotion measures in this clinical trial will include the Self-Rating Anxiety Scale (SAS), Self-Rating Depression Scale (SDS), and Insomnia Severity Index (ISI). After conversion, higher scores indicate severe emotional problems, such as depression, anxiety, and stress.

### Oversight and Monitoring

#### Data Collection and Management

The investigator will prepare original documents for each participant upon their random entry into the study. All pertinent information will be documented in the CRFs weekly and saved in a Microsoft Excel spreadsheet. The fMRI results will be archived on a CD-ROM. To ensure data integrity and quality, an internal data supervision team of researchers, led by the principal investigator, will oversee and audit clinical data. They will regularly or sporadically review the CRFs and promptly provide feedback to researchers in case of any identified issues to prevent the recurrence of similar errors or omissions. It is important to note that all participant data will be strictly confidential and not accessible to the public.

#### Quality Control

Prior to the trial’s commencement, all involved team members will undergo investigator training to acquaint themselves with the administration processes, clarify the procedures for embedding, and become familiar with fMRI [26]. Throughout the trial, each participant will be treated by the same surgeon. The head movement test involving blood oxygenation level–dependent (BOLD) images will be promptly conducted during the scanning process to ensure that head movement remains ≤1.2. If head movement exceeds 1.2, a new BOLD scan will be carried out. Following the scanning, data backup will be performed using both a removable hard disk and CD burning. There will be no changes in assignments, and the migration of individuals between groups will not be allowed. If participants discontinue treatment, the reasons for leaving the study will be recorded.

#### Safety Assessment

Adverse events that occur during treatment and follow-up, such as needle fainting, infection, subcutaneous hematoma, and subcutaneous nodules, will be recorded on the subject’s adverse event record sheet at any time. If a participant develops these adverse reactions, they will be asked to report in detail the symptoms, time of occurrence, duration, and time of abatement. Participants will be closely monitored until all indicators return to a state of complete normalcy. Serious adverse events and unintended events will be reported to the Ethics Committee of Guang’anmen Hospital and relevant authorities promptly in accordance with the regulations. The principal investigator will conduct a cumulative review of all adverse events regularly and may convene meetings with the investigators to assess the study’s risks and benefits if deemed necessary.

#### Modifications

The specific stopping criteria are as follows: (1) adverse reactions or serious complications occurring in patients during clinical treatment; (2) significant errors in the established clinical trial protocols, making it difficult to evaluate the efficacy of the treatment; and (3) significant deviations in the implementation of a well-designed protocol, making it difficult to evaluate the therapeutic effect.

#### Confidentiality

Each participant’s privacy will be maintained to the extent permitted by law. Ombudsmen, inspectors, ethics committees, and regulatory authorities will be granted direct access to participants’ original medical records to verify clinical trial procedures and data to the extent permitted by applicable laws and regulations and without infringing on participants’ privacy. The personal data of the participants will be kept confidential.

#### Amendments

All amendments and adjustments to this protocol must be agreed upon and signed by all study investigators. The amendments will be submitted to the Ethics Committee for approval and updated in the clinical trial registry.

### Statistical Methods

#### Primary and Secondary Outcomes

Continuous variable data following a normal distribution will be expressed using means (SDs), while those not following a normal distribution will be expressed using medians and quartiles. Categorical data will be depicted as component ratios or percentages. Group comparisons for continuous variables will be conducted using independent samples *t* tests and repeated-measures ANOVA (multiple time-point comparisons). Nonparametric data comparisons will be carried out using rank-sum tests. Categorical variables will be compared using chi-square tests or Fisher exact tests. All data analyses and graph representations will be processed using R 4.0.l software (R Project for Statistical Computing). Statistical significance will be defined as *P*<.05.

#### fMRI Examination

Resting-state fMRI data processing and analysis will be conducted using DPABI (Chao-Gan Yan) and SPM12 (Functional Imaging Laboratory) software with the MATLAB platform.

##### Preprocessing

The initial 10 time points will be removed, and corrections will be made for both time and head movements. Data with head movements exceeding translations of >2 mm or rotational shifts of >2° will be excluded. Linear drift will be removed, and regression will be applied to remove interference from white matter, cerebrospinal fluid, and the whole brain signal. Functional images will be registered to T1 gray matter structural images, followed by spatial smoothing with a Gaussian kernel (4-mm half-width height), delinearization, drift correction, and filtering within the range of 0.01-0.1 Hz. All data from participants will be assessed for quality.

##### Methods for Brain Functional Network Connectivity Analysis

The amplitude of low-frequency fluctuation (ALFF) method will be employed to calculate changes in brain-wide low-frequency amplitudes before and after treatment. Brain regions identified in the ALFF results, related to the study hypothesis, will serve as seed points. The average time series of these seed regions and their domain signals will be extracted, and correlations with the remainder of the whole brain will be calculated.

### Ethical Considerations

The study protocol complies with the ethical standards of the Declaration of Helsinki. The Ethics Committee of Guang’anmen Hospital has approved the study protocol (approval number: 2022-114-KY; September 7, 2022), which has been registered in the Chinese Clinical Trial Registry (trial registration number: ChiCTR2200066274; registered on November 30, 2022). The investigators have the responsibility to fully and comprehensively introduce the purpose, procedures, possible risks, and subsidy of this study to the participants and get signatures on 2 informed consent forms, one kept by the participant and the other kept by the researcher, which should be retained as a clinical research document for reference. The participants will receive a subsidy of 300 CNY (US $41.27) through bank transfer after completing treatment and follow-up. All participants will be free to participate in this study and can decide to withdraw at any time. During the study process, the privacy and confidentiality of the participants will be protected. Each participant’s name will be replaced by a serial number and initials. Their personal information will not be disclosed when the paper is published, and the results will be published regardless of whether the research is positive or negative.

## Results

Recruitment began on March 1, 2023. A total of 4-6 participants are expected to be enrolled per month. The recruitment is planned to be completed on March 1, 2025, and we expect to publish our results by the winter of 2025. As of November 1, 2023, we have enrolled 25 participants with CSU.

To compare all the results, including both primary and secondary outcomes, and evaluate the treatment effect, we will use R (version 4.0.1) for the analyses. ANOVA and independent samples *t* tests will be repeatedly performed for determining differences in variables between baseline and posttest in the treatment and control groups and for comparing the effects of the program between the groups. Moreover, we will determine whether there is a significant difference between the groups by judging the rejection range based on a significance level of .05. Drawing upon the outcomes of prior research, we anticipate that when comparing the primary and secondary efficacy indicators across the intervention and baseline periods, both the treatment and control groups will exhibit a downward trend in scores. However, it is expected that the decrease will be more pronounced and significant in the treatment group.

## Discussion

We hypothesize that wheals and itching will show greater improvement in participants receiving active therapy than in those receiving sham treatment. The results of this study will provide new insights into the effects and mechanisms of acupoint stimulation in the treatment of CSU, aiming to reduce the disease burden and economic cost and to contribute to both academia and public health.

ACE, a variant of acupuncture therapy, is based on the theory of acupuncture in TCM and employs absorbable surgical sutures to produce long-lasting acupoint stimulation in the human body [27]. While the specific mechanism of action remains under investigation, it is believed to be associated with improved local circulation, inhibition of the release of inflammatory factors, reduction in apoptosis, and regulation of cellular factors [28]. Currently, ACE is not widely used to treat skin diseases. However, research and evidence-based medicine [29] have demonstrated that ACE is more effective compared to acupuncture and medicine for the treatment of chronic diseases related to the immune system, such as psoriasis [30], allergic rhinitis [31], and ulcerative colitis [32]. Referring to some research studies [33-35] and previous experience, we designed a randomized, double-blind, placebo-controlled trial to observe the therapeutic effect of ACE on CSU by comparing the outcomes before and after treatment. To the best of our knowledge, this is the first study to use ACE for CSU and to observe its clinical efficacy.

This study will employ ACE—an evolved form of acupuncture—as the primary intervention owing to its swift onset, prolonged effects, and improved compliance. We will enroll 108 CSU patients and conduct a randomized, double-blind, sham-controlled study to corroborate the therapeutic benefits of ACE. Additionally, we will use fMRI to delve into the central mechanism of this therapy and validate the hypothesis of modulating the “cerebellum-limbic lobe-reward-somatosensory circuit,” thereby providing robust clinical evidence for the acupuncture treatment of CSU. Compared with previous studies, this study has a larger sample size, less frequency of treatment, and a higher patient compliance rate.

However, this study has certain limitations. On the one hand, this trial is a single-site randomized controlled trial involving a relatively limited number of CSU patients. In our subsequent phase, we aim to embark on a multicenter clinical study to expand the participant pool and thereby enhance the generalizability of our findings. On the other hand, this study requires the enrollment of patients aged 18-60 years with a UAS7 of ≥16. However, this selection criterion may exclude some patients with mild symptoms, patients who can effectively control their condition with medication, and younger or older patients, thereby preventing them from receiving ACE treatment. Furthermore, this study only involves an 8-week intervention for patients, and there may be patients who require a longer period of treatment to achieve significant effects.

If ACE is proven to be effective, it could lead to comparisons with acupuncture or pharmaceutical treatments, offering evidence to support ACE as a novel nonpharmacological approach for CSU management. Furthermore, we can endeavor to integrate modern and traditional techniques to develop a medicinal thread as a potential material for ACE. After the research results are released, we will promote the acupuncture treatment plan for CSU through academic conferences, training courses, standardized training, and other forms of promotion. Moreover, we will promote this project at corresponding community health service centers and support institutions. Through academic exchange, guidance, and article publication, we will promote the academic achievements of this project nationwide, which will facilitate the transformation, promotion, and application of the research results.

## Abbreviations

ACE: acupoint catgut embedding
ALFF: amplitude of low-frequency fluctuation
BOLD: blood oxygenation level–dependent
CRF: Case Report Form
CSU: chronic spontaneous urticaria
CU-Q2oL: Chronic Urticaria Quality of Life Questionnaire
DLQI: Dermatology Life Quality Index
FA: flip angle
fMRI: functional magnetic resonance imaging
FOV: field of view
HPA: hypothalamic-pituitary-adrenal
MRI: magnetic resonance imaging
TCM: traditional Chinese medicine
TE: time to echo
TR: time to repetition
UAS7: urticaria activity score over 7 days
